# Effect of ozone oil and non-surgical periodontal treatment in patients with type 2 diabetes. *In-vivo* and *in-vitro* studies with fibroblasts and *Candida albicans*

**DOI:** 10.1590/1678-7757-2024-0080

**Published:** 2025-01-20

**Authors:** Raquel Alves do CARMO, Ana Carolina Organista CÖRNER, Eduardo Ferreira MARTINS, Gabriel OUVERNEY, Julio Cesar THURLER, Bruno Kaufmann ROBBS, Vinicius D’Avila Bitencourt PASCOAL, Elisa ESPOSITO, Luciane Portas CAPELO, Gabriela Alessandra da Cruz Galhardo CAMARGO

**Affiliations:** 1 Universidade Federal Fluminense Instituto de Saúde de Nova Friburgo Departamento de Clínica Odontológica Nova Friburgo Rio de Janeiro Brasil Universidade Federal Fluminense, Instituto de Saúde de Nova Friburgo, Departamento de Clínica Odontológica, Nova Friburgo, Rio de Janeiro, Brasil.; 2 Universidade Federal de São Paulo Instituto de Ciência e Tecnologia da São José dos Campos São Paulo Brasil Universidade Federal de São Paulo, Instituto de Ciência e Tecnologia da São José dos Campos, São Paulo, Brasil.; 3 Universidade Federal Fluminense Programa de Ciências Aplicadas e Produtos para Saúde Niterói Rio de Janeiro Brasil Universidade Federal Fluminense, Programa de Ciências Aplicadas e Produtos para Saúde, Niterói, Rio de Janeiro, Brasil.; 4 Universidade Federal Fluminense Programa de Pós-Graduação em Biologia Niterói Rio de Janeiro Brasil Universidade Federal Fluminense, Programa de Pós-Graduação em Biologia, Niterói, Rio de Janeiro, Brasil.; 5 Universidade Federal Fluminense Instituto de Saúde de Nova Friburgo Departamento de Ciências Básicas Nova Friburgo Rio de Janeiro Brasil Universidade Federal Fluminense, Instituto de Saúde de Nova Friburgo, Departamento de Ciências Básicas, Nova Friburgo, Rio de Janeiro, Brasil.

**Keywords:** Periodontitis, Periodontal Diseases, Ozone therapy, Fibroblasts, Candida albicans

## Abstract

**Aim:**

To evaluate the clinical effectiveness of ozonated sunflower oil (Oz) as an adjunctive of non-surgical periodontal therapy in patients with type 2 diabetes mellitus (DM2), on fibroblast cell viability and migration and the effectiveness of Oz on a *Candida albicans* (*C. albicans*) culture.

**Methodology:**

In total, 32 sites in 16 DM2 with moderate to advanced periodontal disease with periodontal pocket depths ≥5mm were selected. The treatments were divided into two groups: control, saline solution (SS) as an adjunctive of scaling and root planing (SRP+SS), and test, Oz as an adjunctive of SRP (SRP+Oz). Hematological [fasting glucose level (FGL) and hemoglobin A1c (HbA1c)] and microbiological samples were collected from the participants at baseline and three months after periodontal treatment and the microbiological samples were analyzed by PCR. *C. albicans* was previously tested by the agar diffusion test. The effect of Oz was tested on cell viability and fibroblast migration.

**Results:**

The groups showed no statistically significant differences (paired t-test-p>0.05) regarding hematological parameters, FGL (median - baseline 171.41, 3 months 164mg/dL), and HbA1c (baseline 8%, 3 months 7.5%) (Kruskal-Wallis One-Way Nonparametric-p>0.05) after periodontal therapy. The groups showed statistical differences for periodontal parameters between baseline and three months (paired t-test-p<0.05). PCR analysis showed a reduction in the percentage of *C. albicans* in the SRP+Oz group after three months (McNemar’s test-p=0.002). Cell viability was lower in the high glucose Dulbecco’s modified Eagle’s medium (4500 mg/L) than in low glucose (1000 mg/L) (RM-ANOVA-p<0.0001). The wound healing test showed reduced fibroblast migration (one-way ANOVA with Dunnett’s post-test-p<0.01). Oz showed high *C. albicans* antifungal inhibition (Kruskal-Wallis test-p=0.0001).

**Conclusions:**

SRP+Oz effectively reduced *C. albicans in-vitro* and *in-vivo* but showed no clinical improvements compared to the control. Cell viability and wound healing of fibroblasts showed no improvements.

## Introduction

Diabetes mellitus encompasses a heterogeneous group of disorders that alters glucose tolerance or impairs lipid and carbohydrate metabolism. Type 2 diabetes mellitus (DM2) results from defects in the insulin molecule or from altered cell receptors for insulin and represents impaired insulin function (insulin resistance) rather than deficiency.^1^

The association of periodontitis with diabetes mellitus has been widely studied.^2^ The mechanisms by which diabetes influences the periodontium have been reported: altering hosts’ immune, inflammatory, and wound-healing responses, accumulating advanced glycation end products, and inducing high levels of pro-inflammatory cytokines.^3,4^ Hyperglycemia induces oxidative stress, which is related to the exacerbation of periodontitis and a sustained chronic inflammatory status, and causes dysfunction in immune cells, impairing migration and apoptosis.^3^ Furthermore, surgical or non-surgical periodontal treatment improves glycosylated hemoglobin (HbA1c) control in diabetic patients with poor glycemic control.^4^ Many studies have suggested the bidirectional relation between diabetes and periodontitis as periodontal diseases are more severe and prevalent in diabetic patients. Moreover, periodontitis compromises glycemic control.^3,4^ Thus, periodontal therapy seems to improve metabolic control, and sufficient evidence supports a significant association between periodontal therapy and metabolic control in diabetic patients.^4^

Patients with diabetes show periodontal pathogenic bacteria, such as *Tannerella forsythia, Porphyromonas gingivalis*, and *Treponema denticola*, and higher levels of *Candida* ssp. in periodontal sites.^5,6,7^ Chronic inflammation can affect the gingiva (gingivitis) and when untreated advances to supporting tissues of the teeth (ligament and bone), leading to the development of periodontitis, which destroys tissue and causes alveolar bone loss due to a pathogenic biofilm.^5^ *Candida* spp. is an opportunistic pathogen that causes disease in hosts who are compromised due to underlying local or systemic pathological processes. Infections can be linked to several local and general predisposing factors, such as DM. *Candida* species have often been isolated from the oral cavities of diabetics, with the highest rate of colonization occurring in patients with poor glycemic control.^7,8^

Many therapeutic strategies have been developed to improve periodontal conditions, among which ozone is of particular concern.^9,10^ Ozone is an unstable molecule consisting of three oxygen atoms that quickly break down into oxygen and one oxygen atom that acts as a powerful oxidizing agent that kills microorganisms. Due to its quenching ease, ozone has antioxidant effects in lipophilic and hydrophilic compounds and various antioxidant enzymes.^10-13^ Due to their antiseptic properties (removal of pathogens), ozone and its release oxygen stimulate the growth of fibroblasts and keratinocytes in the wound and produce intercellular matrices that help in wound healing and recovery.^14^ Ozone acts by diverse mechanisms, including direct antimicrobial effects, immunoregulation, antioxidant defenses, among others. Its antimicrobial impact involves disrupting microorganism nucleic acid or liposome shells, increasing membrane permeability for ozone entry.^15^

Studies in periodontics highlight the efficacy of ozone against gram-positive and gram-negative bacteria, viruses, and fungi.^16^ Ozone offers a potential antiseptic alternative in the non-surgical periodontal treatment for diabetic patients, offering non-invasive antimicrobial and immunostimulant effects, accelerating wound healing, significantly reducing saliva metalloproteinase-8 concentrations, and decreasing collagen destruction and damage to the periodontium.^17,18^ Although reports show that ozone can enhance periodontal therapy in patients with periodontitis, no study has evaluated ozonated sunflower oil (Oz) associated with non-surgical periodontal therapy and its effects in people with diabetes or on fibroblasts and *Candida albicans* (*C. albicans*). Despite the good biocompatibility and absence of identified long-term limitations of ozone,^16^ disagreements remain in the literature regarding its effectiveness.^18^

Based on the above, the primary objective of this study was to evaluate the clinical and microbiological responses of Oz as an adjunctive of non-surgical periodontal therapy in DM2 patients. Its secondary objective aimed to analyze the *in-vitro* fibroblast cell viability and migration and the antifungal response to Oz in a *C. albicans* culture.

## Methodology

### Study Design

This is a prospective clinical and laboratory randomized controlled, split mouth, triple blinded study.

### In-vitro experiments

#### Ozone oil characteristics

In this study, ozonized sunflower seed oil was prepared with a peroxide value of 500 to 600 mEq/kg, incorporated in sunflower oil using a portable ozone production device and a bubbling reaction system (ANVISA number: 25351.191000/2022-74). Ozonized oils are produced from two ingredients: vegetable oil and ozone gas. The ozone molecule, when in contact with vegetable oil, results in a chemical reaction in which ozone reacts with the double bonds of carbon in lipid chains and produces new molecules such as ozonizes and peroxides. As it contains a predominance of fatty acids with double unsaturation, sunflower oil is more reactive to ozone, according to Bocci^19^ (2010) (Ozone & Life, Blustratum, São José dos Campos, São Paulo, Brazil).

#### Cell viability (Cytotoxicity) assay

Primary normal human gingival fibroblast was obtained from ATCC (PCS-201-018) and maintained in DMEM (Dulbecco’s modified Eagle’s medium) with high (4500mg/L, Catalog number: 12800017; Gibco; Thermo Fisher, Waltham, MA, USA) or low glucose (1000 mg/L, Catalog number: 11885084; Gibco; Thermo Fisher, Waltham, MA, USA) as indicated, and supplemented with 10% (v/v) FBS (Thermo Fisher, Waltham, MA, USA). The metabolic activity of the cells (viability) was evaluated by the MTT assay, as described by Machado, et al.^20^(2022).

#### Cell Migration Assay (wound healing)

The cell migration assay was performed according to Camargo, et al.^21^(2022). Cells were treated with 0.25, 0.125, 0.06, or 0 uL/mL of Oz in DMEM high or low Glucose, as indicated. Photos were taken under a Zeiss Axio Observer microscope at 0, 24, 48, and 72h and the size of the wound was measured on ImageJ. The percentage of healing was calculated considering the measurement at 0h.

#### Agar diffusion test

The agar diffusion test was performed by experienced microbiologists using Mueller-Hinton agar (Mueller-Hinton agar - Becton, Dickinson and Company^®^, Franklin Lakes, Nova Jersey, USA) discs for antibiogram, with antibiotics for positive control, Amphotericin B, and Nystatin (Nystatin – Sigma Aldrich^®^, San Luis Missouri, USA) as antifungals for positive control and saline solution (SS) 0.09% as negative control, which were tested in *C. albicans* culture (*C. albicans* ATCC 10231). The tests were performed in triplicates.

## In-vivo study

### Randomized controlled split-mouth clinical trial

This study was registered on Clinical Trials NCT05838209. Prior to participation, the purpose and procedures of this study were fully explained to all patients, who signed an informed consent form in accordance with the Declaration of Helsinki. The study protocol (CAAE 89966418.8.0000.5626) was approved by the Ethics Committee at Universidade Federal Fluminense. Individuals of both biological sexes, aged over 18 years, with periodontitis as a manifestation of systemic disease, DM2, severe to moderate periodontitis (Stage II or III), widespread distribution, and a moderate rate of progression (Grade B) according to Caton, et al.^22^(2018) were chosen inclusion criteria. The diagnosis of DM2 was confirmed by an endocrinologist according to criteria previously published by the American Diabetes Association.^23^ Participants were required to have undergone neither periodontal treatment nor professional teeth cleaning for at least one year before the study. Exclusion criteria were hypersensitivity to Oz components and any evidence of systemic factors that could modify periodontal disease, except DM2, and that may directly interfere with the conclusion of the work (bias), such as smokers, osteoporosis types I and II, alcoholism, immunosuppression, any alteration that could modify participants’ periodontal disease profile, and physical/emotional stress drugs that influence periodontal tissues. Pregnant or lactating women, individuals with clinical manifestations of oral candidiasis, or those who had used antibiotics, anti-inflammatories, or hormone replacement therapy in the previous six months were also excluded.

The sample size was based on the study by Patel, et al.^24^(2012), using the mean and standard deviation values of clinical attachment level (CAL). The calculation, considering a 5% significance level and 80% study power, performed on OpenEpi (http://www.openepi.com), indicated a sample size of 32 sites. Therefore, as this is a split-mouth study, 16 volunteers were selected for the study. The allocation of treatments was performed by an experienced clinician (GACGC), using computer software (Microsoft Excel 365, United States, USA).

Hematological parameters were assessed to determine the presence of DM2. Blood samples were collected in vacuum tubes in the morning to determinate fasting glucose levels (FGL) and glycated hemoglobin (HbA1c) at a specialized clinical laboratory in the same municipality (De Vita Laboratory, Nova Friburgo, Rio de Janeiro State, Brazil). The samples were collected prior to periodontal treatment and three months after the treatment.

Characteristics of subjects and biometric parameters: age, sex, weight, height, body mass index, waist circumference, and medical and dental histories were collected prior to periodontal treatment. An experienced periodontist (ACOC) evaluated the clinical parameters and collected samples of the biofilms from the periodontal pockets. The following periodontal parameters were evaluated: plaque index (PI), presence or absence of bleeding on probing (GI), periodontal pocket depth (PPD), gingival recession (GR), and clinical attachment level (CAL), recorded in millimeters, using a periodontal probe PCP15 (PCP-UNC15, Hu-Friedy, Chicago, IL, USA) at six sites, excluding third molars. Sites with a PPD >5mm in at least two teeth in different sextants were selected and labeled to receive subgingival treatment and the collection of samples. The intra-examiner agreement of the categorical variables (PI, GI) using the kappa calculation at the tooth level equaled 0.75. Reproducibility of continuous variables (PPD, GR, and CAL) totaled 0.70, as evaluated by the intraclass correlation coefficient. The examiner (ACOC) had no access to previous records.

Overall, two treatments at randomly chosen sites were separated into two groups: Oz (Ozone & Life, Blustratum, São José dos Campos, São Paulo, Brazil), used as an adjunctive of scaling and root planing (SRP) (SRP+Oz) and SS 0.09%, as a negative control, adjunctive of SRP (SRP+SS). Ultrasonic scalers (Dabi Atlante, Rio de Janeiro, RJ, Brazil) and Gracey curettes (Hu-Friedy^®^, Chicago, IL, USA) were used to remove all calculus and biofilms during SRP. The substances were numbered in syringes labeled 1 and 2 for blinded applications and 1 ml of each substance was administered in the previously selected sites. After removing baseline samples of biofilm, the non-surgical periodontal treatment (SRP) was performed using ultrasonic devices and manual Gracey curettes (Hu-Friedy^®^, Chicago, IL, USA). SRP was scheduled in one session in which the substances were applied. To avoid carry-across (interference between treatments), the sextants were isolated with a cotton roll and a sterile curette was used for each sextant. This is a triple blinded study. The participants, investigator (ACOC), and statistician (GACGC) did not know which treatment or intervention participants received until the end of the clinical trial.

Another clinician (RAC) conducted the periodontal intervention and substance applications. After treatment, the clinician provided standardized oral hygiene instructions to be followed at home, together with the modified Bass tooth-brushing technique and interdental cleaning with dental floss twice a day. Neither antibiotics and anti-inflammatory drugs were prescribed after the periodontal intervention nor were subjects guided to use them. Instructions for oral hygiene and prophylaxis were administered at 30-, 60-, and 90-day intervals. After three months of follow-up, the hematological and clinical parameters were reevaluated, and samples of biofilm were collected from the two selected sites. The data were recorded to compare the conditions before and after treatment. This study ended after clinical data collection three months after its interventions. No side effects were reported by participants.

### PCR analyses for C. albicans

Biofilms were collected prior to SRP and three months after the periodontal treatment from the same sites. The biofilm was collected using sterile Gracey curettes in Eppendorf tubes containing Tris-EDTA and stored at −20^º^C. Samples were analyzed microbiologically by polymerase chain reaction (PCR) to identify the presence of *C. albicans* before and after periodontal treatment to determine the effectiveness of the intervention on the percentage of *C. albicans* in patients with DM2. The primer to *C. albicans* and the universal primer 16S have been described by Sardi, et al.^6^(2011). Tests were performed to verify primer specificity. PCR reactions with specific primers for *C. albicans* were processed for this. The PCR conditions had been published by Barros, et al.^25^(2008).

## Statistical analysis

Statistical tests were performed on Statistix (Analytical Software, Tallahassee, USA, Version 8, 2003) for clinical, hematological, and microbiological *in-vivo* parameters. The Shapiro-Wilk test was used to check variable normality. Prism (Graphpad Software, San Diego, CA, USA) was adopted for *in-vitro* tests, agar diffusion, and cell viability and migration.

The agar diffusion test used a non-parametric test (Kruskal-Wallis test). The McNemar’s test was used for the frequency of sites with *C. albicans* between the SRP+SS and SRP+Oz groups and the two-time intervals (baseline and three months). Cell viability and cell migration used repeated measures analysis of variance (RM-ANOVA) and one-way ANOVA with Dunnett’s Post-test.

The variable “age” was parametric and used two-sample t tests. The variable “sex” used a binomial test. For skin colors, X^2^ adjustment was used. The Kruskal-Wallis one-way nonparametric AOV was used for hematological parameters (FGL and HbA1c). The paired t-test was used to compare periodontal parameters (PI, GI, PPD, GR, and CAL), which were parametric variables between baseline and three months and between SRP+SS and SRP+Oz groups. Statistical significance for all variables was defined at 5%.

## Results

### In-vitro experiments

#### Cell viability (Cytotoxicity) assay

**Figure 1:**
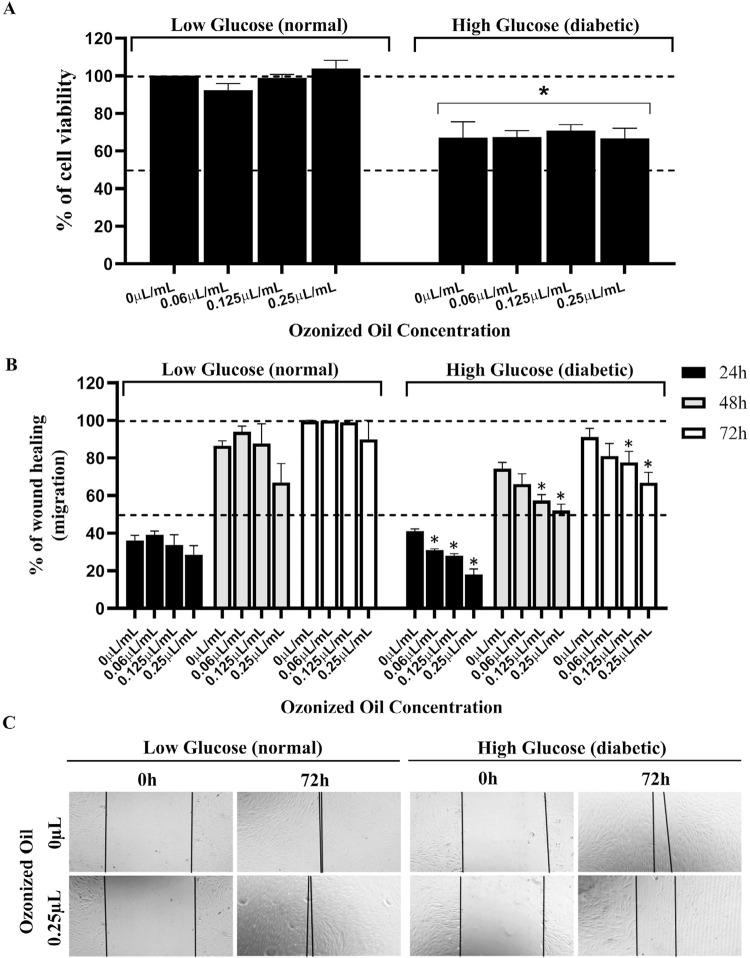
Influence of Oz on cell viability and migration. Primary human gingival fibroblasts were used in all experiments. Cells were grown in DMEM with low (normal phenotype of glucose concentration; 1000 mg/L of glucose) or high glucose (mimicking diabetic phenotype; 4500 mg/L of glucose) where indicated. A) Cell viability assay: Columns indicate the percentage of cell viability obtained by MTT assay, of each concentration of Oz tested after the 48-h treatment. Results were calculated in relation to untreated control at normal glucose concentration (0µL/mL of oil; low glucose). *Statistically significant difference between low and high glucose groups (RM-ANOVA (p<0.0001). B and C) Wound healing test (cell migration): Plated cells were accompanied until reaching confluency. After scratching (wound generation), cells were treated with the indicated Oz concentration and the size of the wound was measured by microscopy at 0, 24, 48, and 72 hours. B) Graph showing the % of healing in relation to 0 h of each treatment. C) Representative microscopies of B. Horizontal lines represent the wound borders. All experiments were repeated at least three times. One-way ANOVA with Dunnett’s Post-test was performed for A and B (*p<0.01).

Oz caused no significant alterations in cell viability at any tested concentration (Figure 1A). However, reduced cell proliferation occurred in the medium with a high glucose concentration in comparison with cells grown in the normal glucose condition (low glucose) (RM-ANOVA - p<0.0001). This result shows that Oz failed to interfere in cell proliferation as opposed to glucose concentration.

#### Cell Migration Assay (wound healing)

Wound healing (cell migration) seems to respond negatively to Oz in a dose dependent manner (Figure 1B-C). Non-treated cells (0 µL/mL) of both normal and diabetic phenotype mediums showed a similar capability for migration. However, Oz significantly reduced (one-way ANOVA with Dunnett’s Post-test - p<0.01) wound healing in a dose-dependent manner in the presence of diabetes and tended (but with no statistical significance) to decrease in normal conditions. Although migration supported no clinical use of Oz, the periodontal intervention applied it in hopes of a different response in patients (Figure 1).

Together, these data from the cell viability and migration assays support the finding that diabetes impairs wound healing by possibly interfering cell migration and proliferation and shows that Oz fails to aid these processes, significantly impairing the migration capability of the fibroblasts in patients living with diabetes.

#### Agar diffusion test

**Figure 2:**
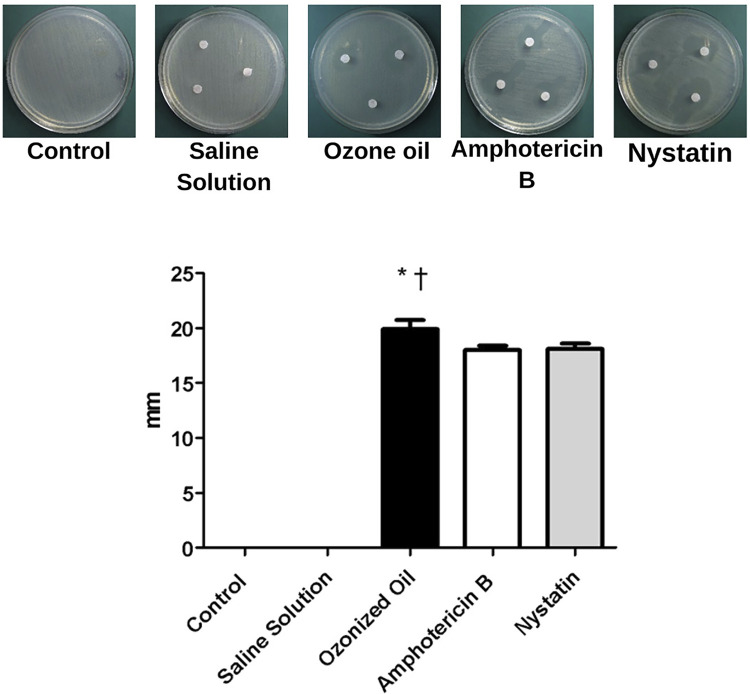
Agar diffusion test for *C. albicans* (ATCC 10231).

The agar diffusion test showed higher mean values for Oz than the untreated treatment and negative control, the values of which statistically and significantly differed from the untreated (p=0.0001) and negative SS controls (p=0.0001) (Kruskal-Wallis test). The inhibited growth zone diameters between Nystatin and Amphotericin B (positive controls) and Oz showed no statistically significant differences. These results evince the antifungal effects of Oz against *C. albicans* and show that Oz can inhibit *C. albicans* in clinical periodontal practice (Figure 2).

*Statistically significant difference between control and Oz (Kruskal-Wallis test – p=0.0001).

^†^Statistically significant difference between SS and Oz (Kruskal-Wallis test – p=0.0001).

## In-vivo study

### Randomized controlled split-mouth clinical trial

This study included 32 periodontal sites of 16 DM2 uncontrolled patients, with a mean age of 57.37±10.05 years, seven men and nine women, from the graduate clinic in periodontology, School of Dentistry, Fluminense Federal University (Nova Friburgo, RJ, Brazil) from December 2019 to March 2020.

This study found no statistically significant differences three months after the periodontal intervention in subjects’ age (p=0.5) and sex (p>0.05). However, skin color was higher for white people (75%, when compared to 6.3% of Black and 18.8% of Brown individuals) with a statistically significant difference (*X*^2^adjustment - p=0.015). The hematological parameters in DM2 patients, fasting glucose (median - baseline 171.41 and three months 164mg/dL) and HbA1c (baseline 8% and three months 7.5%) showed no statistically significant differences between baseline and three months (Kruskal-Wallis One-Way Nonparametric AOV - p>0.05). Results confirm that topically applying Oz failed to change the systemic disorder or biometric and hematological parameters. Patients were overweight and had uncontrolled glycemia (Tables 1 and 2).

**Table 1:** Characteristics (mean±SD) of DM2 patients at baseline and 3 months after periodontal treatment.

	DM2 group (n=16)	*p value*
**Age (years)**		
Baseline	57.37±10.05	0.5000
3 months	57.37±10.05	
**Sex**		
Male	7 (43.8 %)	1.000
Female	9 (56.3%)	
**Skin colors**		
White	12 (75%)^§^	0.015
Black	1 (6.3%)	
Brown	3 (18.8%)	

^§^Statistically significant difference between skin colors (X^2^ adjustment - p≤0.05).

**Table 2:** Hematological parameters of DM2 patients - median (maximum-minimum) at baseline and 3 months after periodontal treatment.

	DM2 group (n=16)	*p value*
**FGL (mg/dL)**		
Baseline	171.41 (103 – 263)	1.000
3 months	164 (122.63 – 263.26)	
**HbA1c (%**)		
Baseline	8 (5.2 – 10.8)	0.6560
3 months	7.5 (5.9 – 10.8)	

After non-surgical periodontal treatment, this study found no statistically significant differences in periodontal parameters between SRP+Oz and SRP+SS groups (paired t-test - p>0.05). Both groups showed statistical differences (paired t-test - p<0.05) between baseline and three months for GI, PPD, and CAL (Table 3).

**Table 3:** Periodontal parameters (mean±SD) of DM2 patients for SRP+SS and SRP+Oz at baseline and 3 months.

	SRP + SS	SRP + Oz	*p value*
	**(n=16)**	**(n=16)**	
**PI (%)**			
Baseline	55.7±40.69	56.24±44.25	0.946
3 months	35.41±42.98	35.41±46.69	1.000
*p value* (within groups)	0.177	0.086	
**GI (%)**			
Baseline	45.83±31.91	54.16±36.76	0.531
3 months	17.7±22.33	11.45±27.7	0.394
*p value* (within groups)	0.006*	0.006*	
**PPD (mm)**			
Baseline	5.2±0.68	5.75±1.73	0.178
3 months	4.18±0.83	4.75±2.01	0.351
*p value* (within groups)	0.002*	0.003*	
**GR (mm)**			
Baseline	0.5±1.41	0.31±0.87	0.188
3 months	0.5±1.41	0.31±0.87	0.188
*p value* (within groups)	n.a.	n.a.	
**CAL (mm)**			
Baseline	5.75±1.87	6.06±2.29	0.352
3 months	4.68±1.78	5.06±2.29	0.535
*p value* (within groups)	0.002*	0.003*	

*Statistically significant differences between baseline and 3 months (Paired t-Test - p<0.05). n.a.: not applicable.

This research measured PI and GI (Table 3) to evaluate the percentage of plaque on all tooth surfaces and bleeding on probing at two time intervals (baseline and three months) (paired t-test) and found no significant differences between the SRP+SS and SRP+Oz groups (paired t-test). Both treatment groups showed reduced PI and GI after three months and improved oral hygiene habits. Concerning the prevalence of gingivitis, the two groups showed no differences. The groups seemed to respond similarly regarding oral hygiene habits based on PI changes but the median GI was lower in the SRP+Oz group after three months, despite no statistical differences between the test and control group.

Table 3 also shows significant reductions in PPD and CAL (paired t-test) after three months for the SRP+SS and SRP+Oz groups. However, no significant differences occurred between groups (paired t-test). GR showed no changes between baseline and three months, and the two groups showed no significant statistical differences for GR (paired t-test).

### PCR analyses for C. albicans

**Figure 3:**
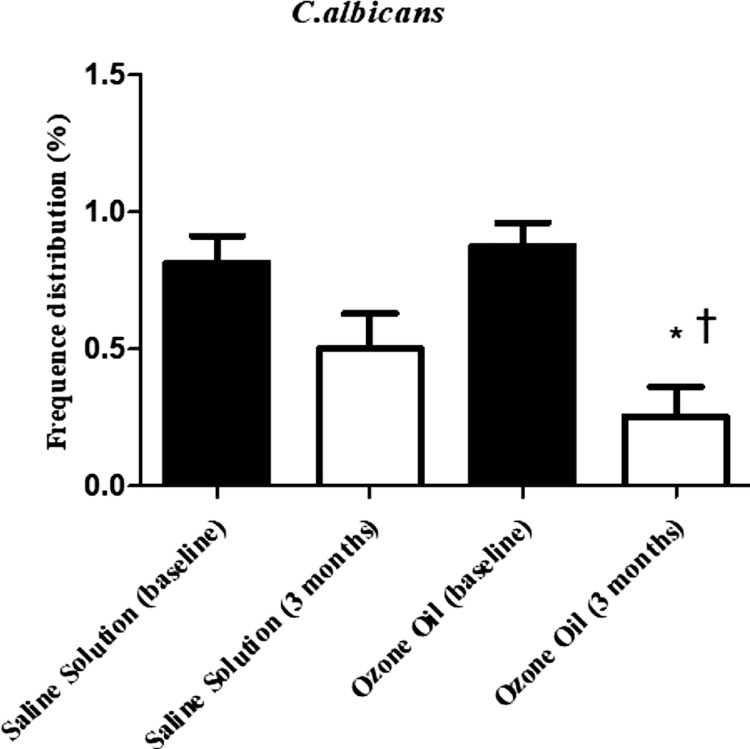
Frequency distribution of *C. albicans* at baseline and three months in SRP+SS and SRP+Oz Groups after PCR analysis.

Figure 3 shows the frequency distribution of *C. albicans* for the SRP+SS and SRP+Oz groups at baseline and three months. The percentages of sites with *C. albicans* at baseline equaled 81.3% for SRP+SS and 87.5% for SRP+Oz groups. after three months, these percentages decreased to 50 and 25%, respectively. SRP+SS and SRP+Oz showed no significant differences at baseline (McNemar’s test - p=0.317), doing so at three months (McNemar’s test - p=0.046). A statistically significant difference between baseline and three months occurred only for the SPR+Oz group (McNemar’s test - p=0.002). The PCR results confirmed that Oz can reduce the percentage of *C. albicans* after three months of follow-up of non-surgical periodontal therapy in patients with DM2 (Figure 3). These results provide evidence of the antifungal activity of Oz when associated with non-surgical periodontal therapy.

*Statistically significant difference between baseline and three months for SRP+Oz (McNemar’s test - p=0.002).

^‡^Statistically significant difference at three months between SRP+SS and SRP+Oz (McNemar’s test - p=0.046).

## Discussion

This study aimed to test Oz as an adjunctive of SRP in DM2 patients. Initially, *in-vitro* testing was performed of the viability and migration of fibroblasts and the antifungal effect on *C. albicans.* Oz was effective for inhibiting *C. albicans in-vitro* and *in-vivo*, although the periodontal clinical results did not show a significant difference between SPR+SS and SRP+Oz. Both groups showed a reduction in periodontal parameters three months after the periodontal treatment.

Diabetes constitutes a systemic condition that is considered a risk factor for periodontal disease and that impairs the healing of acute wounds.^26-28^ This study evaluated the effects of Oz on wound healing (cell migration) under high glucose conditions. Some studies show that a high glucose concentration in the medium reduced fibroblast proliferation by almost 40% in comparison to cells grown in a normal glucose condition (low glucose),^29,30^ whereas another study found no significant differences between control and ozone-treated cells in the scratch assay.^12^ A considerable increase in fibroblast migration occurred in cells treated with 8 μg/mL of ozonized saline when compared to control, although the migration assay in the diabetic condition showed a delay in fibroblast healing, suggesting that Oz failed to accelerate healing. This study used Oz in a high glucose medium to improve wound healing, although it seems to respond negatively to Oz dilutions (0.25, 0.125, 0.06, or 0 uL/mL) in a dose-dependent manner. Diabetes conditions impaired the wound healing process by possibly interfering in both migration and proliferation of fibroblasts, whereas Oz failed to increase healing and impaired fibroblast migration. Although the cell migration findings failed to benefit from the Oz dilutions in this study due to the lack of improvement in fibroblast migration of wound healing,^31^ applying them during the periodontal intervention showed the benefits of the Oz association to promote periodontal healing and reduce clinical periodontal parameters. The effect of Oz remain controversial in the literature and more *in-vitro* studies are needed to understand the effect of Oz on wound healing.

Ozone works positively by destroying bacteria, fungi, and viruses. The antimicrobial effect of ozone results from its action on cells, damaging the cytoplasmic membrane due to ozonolysis of dual bonds and modifying intracellular contents (oxidation of proteins, loss of organelle function), from secondary oxidant effects. This action is non-specific and selective to microbial cells; it damages no human body cell because of their major antioxidative ability. Ozone is very efficient in antibiotic resistant strains and its antimicrobial activity increases in liquid environments with an acidic pH. In viral infections, the ozone action lies in the intolerance of infected cells to peroxides and its change in the activity of reverse transcriptase, which takes part in the synthesis of viral proteins.^14,32^

The ozone molecule has a very strong oxidizing ability, which means it can break down molecules into their component parts.^14,32^ Sunflower oil is also effective against gram-positive bacteria, such as *Pseudomonas aeruginosa* and *Escherichia coli.* When applied topically, sunflower oil effectively treated methicillin-resistant *Staphylococcus aureus*.^33^ Sunflower oil has also effectively treated *Acinetobacter bronchitis*, a lung infection due to *Acinetobacter baumannii* and against *C. albicans, a* yeast infection.^33^ This study agrees with these findings as Oz could inhibit *C. albicans in-vitro* and *in-vivo*, suggesting that it was effective in both non-diabetic individuals and in DM2.

The characteristics of the DM2 patients suggest that the population in this study had uncontrolled glycemic indices and the non-surgical periodontal therapy failed to improve glycemic control after three months. Periodontal disease is hard to treat and control in uncontrolled diabetic patients as their altered inflammatory and wound-healing responses impact periodontal recovery. Moreover, patients with DM2 are more susceptible to showing periodontal disease.^3,4^ A reduction in inflammation benefits the oral health of DM2 patients and can impact glycemic control.^4^ In this study, after the periodontal treatment, the main indicator of gingival inflammation (GI^22^) showed similar mean values, which were lower than in other studies with DM2 patients, which reported a final result of 25.11% for GI^6^, in comparison to 17.7 and 11.45% for SRP+SS and SRP+Oz, respectively.

The use of ozone associated with SRP remains controversial in non-diabetic patients in the literature. Some authors report that SRP followed by ozone therapy fails to contribute to additional improvement in periodontal clinical parameters in patients with periodontal disease^9,12,18^. However, other authors report that ozone can promote additional effects of SRP^17,32,34-36^. For people with diabetes, no studies exist regarding non-surgical periodontal therapy using Oz or ozone therapy, indicating that this topic should be further studied. It is important to consider the way that ozone is administered, such as in water, oil, or gas, and the ability of operators to introduce it at the depth of the periodontal pocket, which could influence results. Many applications of ozone can delay or alter periodontal healing.^37^ Thus, this study used a syringe to introduce Oz into the periodontal pockets in one application. The principal benefit of the use of ozone associated with non-surgical periodontal therapy refers to its antimicrobial properties. Ozone has effectively treated gram-positive and gram-negative bacteria, viruses, and fungi.^38^ Nagayoshi, et al.^39^(2004) treated periodontopathogenic bacteria *in-vitro* with ozone water and reported effectiveness against Streptococcus, *Porphyromonas gingivalis* and *endodontalis, Aggregatibacter actinomycetemcomitans,* and *C. albicans* in culture and in biofilms. The authors concluded that Gram-negative anaerobes, such as *Porphyromonas endodontalis* and *Porphyromonas gingivalis* were substantially more sensitive to ozonated water than gram-positive oral streptococci and *C. albicans* in pure culture. Our agar diffusion test results agree with these authors as they showed statistically significantly different *C. albicans* results from control with higher efficiency, such as antibiotic inhibitions.

This study showed reductions in PI, GI, PPD, and CAL three months after treatment and the PCR analyses showed reduced percentages of *C. albicans* after non-surgical periodontal treatment in patients with DM2. Some authors have observed higher percentage reductions in the PI and GI using ozone irrigation in comparison to chlorhexidine.^17^ Using ozone appreciably reduced periodontopathogen percentages, such as those *Actinobacillus actinomycetemcomitans (A.a.)*, when compared to no change in *A.a.* occurrence with chlorhexidine. The antifungal effect of ozone from baseline to the seventh day was pronounced during the study period, unlike chlorhexidine, which showed no antifungal effect.^17^ Moreover, the topical gaseous ozone applications showed statistically significant improvements in clinical parameters, decreasing anaerobic species^36^ and the amount of bacteria since *T. forsythia, T. denticola, and Fusobacterium nucleatum* in adult periodontal patients decrease or disappear when ozonated water is associated with the conventional periodontal treatment.^35^

Sardi, et al.^6^(2011) justified the use of Oz in DM2 as they found the strong colonization of *Candida spp.* in the periodontal sites of patients with diabetes when compared to non-diabetic individuals. Moreover, patients with diabetes show generalized chronic periodontitis with a higher prevalence of *C. dubliniensis* followed by *C. albicans. Candida spp.* is considered an opportunistic pathogen that causes disease in hosts who are compromised due to underlying local or systemic pathological processes. *Candida spp*. shows several important virulence factors, such as proteolytic and hemolytic activity, and the ability to adhere to epithelial cells and penetrate connective tissues, causing inflammatory reactions,^40^ and may be detrimental to the periodontal environment. However, the role of yeasts, mainly *C. albicans,* in periodontitis remains unclear.

These results suggest caution when analyzing the results of this study. It has some limitations regarding time evaluations, Oz concentration in the gingival fluid, and its substantivity. Furthermore, this study included no group without DM2 and had a limited sample size. This study suggests that future research monitor patients for a longer time and find the release and concentration of Oz in the crevicular gingival fluid during periodontal healing. Oil concentration (500 to 600 mEq/kg) agreed with the manufacturer’s recommendations. Finally, further studies with a larger number of patients and better analysis of the Oz concentration are needed to verify if these results are maintained or could be improved.

## Conclusion

SRP+Oz was effective for reducing *C. albicans* in DM2 patients three months after periodontal therapy and showed the antifungal effects of *C. albicans in-vivo* and *in-vitro*, but did not improve cell viability and migration of fibroblasts in high glucose medium after *in-vitro* analyses. However, SRP+Oz showed no improvements in periodontal parameters compared to the control group (SPR+SS) after three months of follow up.

## Data Availability

All data generated or analyzed during this study are included in this published article
